# 

**Published:** 1882-09-20

**Authors:** 


### TABLE OF CONTENTS.

ORIGINAL AND SELECTED ARTICLES.

Otalgia from Reflex Dental Irritation, by A. G. Hobbs, M. D., Professor of Eye, Ear and Throat Diseases, in Southern Medical College, Atlanta, Ga., 321. A Case in Practice, by R. L Hinton, M. D., Prescott, Ark., 323. The Differential Diagnosis of the Cause of Sudden Unconsciousness, by R. O. Beard, M. D., 326. Pulsatilla in Inflammation of the Testes, by G. Frank Lydston, M. D., 330. On the Treatment of Chronic Dysentery by Voluminous Enemata of Nitrate of Silver, by Stephen MacKenzie, M. D., F. R. C. P., Physician to, and Lecturer on Medicine, at the London Hospital, 333. An Interesting Obstetrical Experience, 335. On the Treatment of Eczema by Bantingism, by Balmanno Squire, M B, London, 336. A Laboratory Study of Listerine, by Frank M. Deems, M. D., Ph. D., President of Augusta, Ga., Academy of Medicine, etc., 337. A New Method of Embalming Bodies and Preserving Tissues, 339.

ABSTRACTS AND GLEANINGS.

Mangifera Indica, or Mango, 340. Surgery and the Doctrine of Evolution, 341. Colostrum in the Milk as a cause of Cholera Infantum, 342. Pasteur's Investigations on Rabies, 344. Treatment of Snake-bite; A Dose of Quicksilver, 345. Salicylate of Potassa in Acute Rheumatism and Dyspepsia, 346. The Secrets of our Patients; Does Quinia Increase or Lessen Intracranial Blood Supply, 347. The Anti-Fat Dietary, 348. Intermittent Fever and Quinine Hypodermically; Immunity of the Chinese from Disease; Carbolized Nerves as Ligatures, 349. Castor Oil as a Lactigogue; The Value of Viscum Album or Mistletoe in Affections of the Heart; Large Dose of Chloral, 350.

SCIENTIFIC ITEMS.

Canals on the Planet Mars; The results of the Tenth Census, 351. The "Smartness" of Worms; In Round Numbers; Greatness; Cleans Pens, 352.

PRACTICAL NOTES AND FORMULÆ.

Excoriated Papular Eczema; Camphorated Chloral-Tannate of Iodine, 353. Cracked Nipples; Diphtheria, 354. Constipation; Fuming Inhalation in Asthma; Blackberry Extract in Diarrhœa, A New Remedy, 355.

EDITORIALS AND MISCELLANEOUS.

Southern Medical College, Atlanta; Medical College Advertisements; Omission; Mistake, 356. Medical Society of Virginia; American Medical Association, 357. An Art and Industrial Exhibition in the Capitol at Washington, D. C.; Transactions of the Michigan State Medical Society, 358. Book Notices, 359. Receipted; Special Notices, 360.

